# A systematic review of non-randomised evaluations of strategies to improve participant recruitment to randomised controlled trials

**DOI:** 10.12688/f1000research.22182.1

**Published:** 2020-02-05

**Authors:** Heidi R Gardner, Loai Albarquoni, Adel El Feky, Katie Gillies, Shaun Treweek

**Affiliations:** 1 Health Services Research Unit, Health Sciences Building, University of Aberdeen, Aberdeen, Scotland, AB25 2ZD, UK; 2 Institute for Evidence-Based Healthcare, Faculty of Health Sciences and Medicine, Bond University, Gold Coast, Queensland, Australia

**Keywords:** Clinical trial, Participant recruitment, Recruitment interventions, Participant selection, Recruitment strategies

## Abstract

**Background**: Recruitment to trials can be challenging. Currently, non-randomised evaluations of trial recruitment interventions are rejected due to poor methodological quality, but systematic assessment of this substantial body of work may inform trialists’ decision-making about recruitment methods. Our objective was to quantify the effects of strategies to improve participant recruitment to randomised trials evaluated using non-randomised study designs.

**Methods**: We searched relevant databases for non-randomised studies that included two or more interventions evaluating recruitment to trials. Two reviewers screened abstracts and full texts for eligible studies, then extracted data on: recruitment intervention, setting, participant characteristics, number of participants in intervention and comparator groups. The ROBINS-I tool was used to assess risk of bias. The primary outcome was the number of recruits to a trial.

**Results**: We identified 92 studies for inclusion; 90 studies aimed to improve the recruitment of participants, one aimed to improve the recruitment of GP practices, and one aimed to improve recruitment of GPs. Of the 92 included studies, 20 were at high risk of bias due to confounding; the remaining 72 were at high risk of bias due to confounding and at least one other category of the ROBINS-I tool. The 20 studies at least risk of bias were synthesised narratively based on seven broad categories; Face to face recruitment initiatives, postal invitations and responses, language adaptations, randomisation methods, trial awareness strategies aimed at the recruitee, trial awareness strategies aimed at the recruiter, and use of networks and databases. The utility of included studies is substantially limited due to small sample sizes, inadequate reporting, and a lack of coordination around deciding what to evaluate and how.

**Conclusions**: Careful thought around planning, conduct, and reporting of non-randomised evaluations of recruitment interventions is required to prevent future non-randomised studies contributing to research waste.

**Registration**: PROSPERO CRD42016037718

## Abbreviations

CINAHL, Cumulative Index to Nursing and Allie Health Literature; CONSORT, Consolidated Standards of Reporting Trials; EMBASE, Excerpta Medica database; GRADE, Grading of Recommendations Assessment, Development and Evaluation; MEDLINE, Medical Literature Analysis and Retrieval System Online; MRC, Medical Research Council; PRISMA, Preferred Reporting Items for Systematic Reviews and Meta-Analyses; RCT, randomised controlled trial; ROBINS-I, Risk Of Bias in Non-randomised Studies – of Interventions; SWAT, study within a trial

## Introduction

Randomised controlled trials (RCTs) are at the core of evidence-based healthcare. They use random assignments to allocate participants to treatment groups, and therefore guard against selection bias ^1^, whether these involve medicinal products, devices or services. Recruiting participants can be difficult, as can the process of recruiting clinicians to work on the trial with and on behalf of the trial team ^2^.

One important source of evidence for trialists looking for rigorously evaluated evidence on how to effectively recruit participants to trials is the 2018 Cochrane systematic review of interventions to improve trial recruitment ^3^. Despite having no date or language restrictions and including 72 recruitment comparisons, just three are supported by high-certainty evidence ^3^.

This systematic review reported here uses a similar process to the 2018 Cochrane systematic review of interventions to improve trial recruitment ^3^, but with one substantial difference. This review focusses *only* on recruitment interventions that are evaluated using non-randomised methods. Until now, systematic reviews of non-randomised studies of recruitment interventions have been scarcely undertaken due to the perception that non-randomised studies are individually, of low methodological quality. However, the systematic evaluation of a substantial amount of research activity is necessary and worthwhile; without collation, this body of evidence is currently being ignored, and may hold substantial/promising undiscovered effects. Whether evidence of benefit is found for one or more interventions, the trials community will benefit from knowing the outcome of this review. Moreover, aggregating data from non-randomised studies using the Grading of Recommendations Assessment, Development and Evaluation (GRADE) approach ^4^, may raise confidence in the overall body of evidence, and supplement the evidence-base from randomised studies.

## Objective

We conducted a systematic review of non-randomised studies that evaluated the effects of strategies to improve recruitment of participants to RCTs.

## Methods

The full protocol for this review has been previously published ^5^ and registered with PROSPERO ( CRD42016037718). No amendments have been made to the protocol since its publication. A brief summary of methods is given below.

### Types of studies

Non-randomised studies of two or more interventions to improve recruitment to a randomised trial. ‘Non-randomised studies’ are defined as any quantitative assessment of a recruitment intervention that did not randomly allocate participants to intervention or comparison groups. No additional eligibility criteria (e.g. publication year, status, language or journal) were applied.

### Types of participants

Individuals enrolled in a trial. The context of the trial is likely to be healthcare but may not be, for the reason that interventions that are effective in other fields may also be applicable to settings in the healthcare environment.

### Types of intervention

Any intervention or approach aimed at improving or supporting recruitment of participants nested within studies performed for purposed unrelated to recruitment.

### Types of outcome measures

Primary: Number of individuals or centres recruited into a trial.

Secondary: Cost of using the recruitment intervention per trial participant.

### Search methods for identification of studies

We searched the following electronic databases without language restriction for eligible studies: Cochrane Methodology Register (CMR), Medical Literature Analysis and Retrieval System Online (MEDLINE), MEDLINE In-Process, Excerpta Medica dataBASE (EMBASE), Cumulative Index to Nursing and Allied Health Literature (CINAHL), and PsycINFO. The full search strategy is published and freely accessible ^5^. Reference lists of relevant systematic reviews (e.g. 3) and included studies were hand-searched.

The literature searches were carried out between 16 ^th^ October and 11 ^th^ November 2015. On 2 ^nd^ August 2018 an updated search was made in all databases, and a further 2,521 abstracts were found. 460 abstracts from 2018 were screened in duplicate, which led to 10 full texts being checked for inclusion. The ten full texts detailed ten studies, none of which provided sufficient detail about the design or implementation of interventions to allow us to pool data. Adding these studies into the review would not strengthen or disprove the conclusions we had already drawn. For this reason, we have chosen not to carry out a full updated search, and all data presented in this paper reflect the full literature searches carried out in 2015.

### Selection of studies

Two reviewers (HRG and one other) independently screened the abstracts of all search records. Full texts of potentially eligible abstracts were then independently reviewed by HRG and one other to determine inclusion. Disagreements were resolved through discussion.

### Data management and extraction

**Figure 1.**
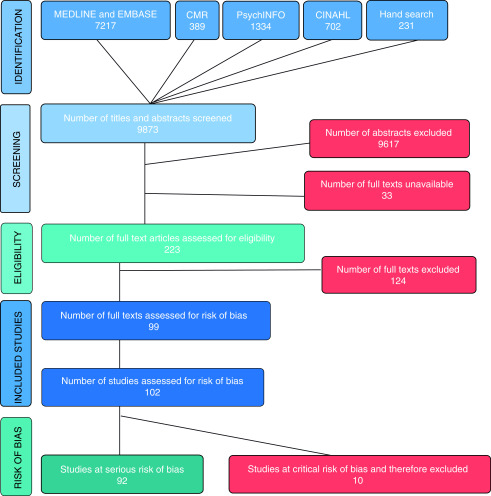
PRISMA flowchart of systematic review (based on 8).

Search results were merged, duplicate records removed, and a master spreadsheet was used to track all inclusions/exclusions to allow us to create a Preferred Reporting Items for Systematic Reviews and Meta-Analyses (PRISMA) flow diagram ( Figure 1). Data were extracted by two reviewers independently (HRG and one other) and collected on specially designed forms (Extended Data File 1 Blank data extraction form 6). Disparities were resolved through discussion.

### Assessment of risk of bias in individual studies

Two members of the project team used the ROBINS-I tool ^7^ to assess studies for aspects of methodological quality such as confounding, participant selection, intervention measurement, departures from the intended intervention, missing data, outcome measurement and selection of the reported result. As per ROBINS-I guidance, studies at critical risk of bias were excluded from any synthesis.

### Analysis

Studies were analysed according to the type of intervention used; interventions were grouped when their form or content was deemed sufficiently alike. We planned to further categorise studies by participant if we found the same intervention applied to more than one type of participant (e.g. patients, staff at recruiting centres).

### Dealing with missing data

Attempts were made to contact study authors to obtain missing data. Analyses were conducted on an intention-to-treat basis where possible; alternatively, data were analysed as reported.

### Assessment of heterogeneity

The nature of the included studies meant that much of the analysis was anticipated to be narrative. Where population, intervention and outcome were sufficiently similar to allow for a meta-analysis, we planned to look for visual evidence of heterogeneity in forest plots, and statistical evidence of heterogeneity using the chi-square test for heterogeneity and the degree of heterogeneity quantified using the I ^2^ statistic ^9^. Where substantial heterogeneity was detected (I ^2^ ≥ 50 %), we planned to investigate possible explanations informally and summarise data using a random-effects analysis where appropriate.

### Assessment of reporting bias

We planned to investigate reporting (publication) bias for the primary outcome using a funnel plot where 10 or more studies of the same population, intervention and outcome were available.

## Results

### Screening and identification of studies

We screened a total of 9,642 abstracts identified by the database search, and 231 articles found through hand searching of review article reference lists. Of the screened abstracts, 256 were suitable to assess for inclusion at full-text stage. We were unable to obtain the full text of 33 of the 256 articles (details in Extended Data File 2 References to studies awaiting assessment ^6^). Of the 223 full-text articles assessed, 124 were excluded; this includes seven articles which required additional data to allow for inclusion (details of excluded studies in Extended Data File 3 Characteristics of excluded studies ^6^). A total of 99 full texts were included, which comprised 102 individual studies; 92 of these were considered to be at serious risk of bias while ten were considered to be at critical risk of bias. The latter group were excluded from the study as per ROBINS-I guidance (see risk of bias assessments for these studies in Extended Data File 4 Studies that were at a critical risk of bias and therefore excluded from this review ^6^).

### Description of studies

Of the 92 included studies, 90 studies assessed interventions that aimed to improve the recruitment of participants to trials (55123 individuals and 172 couples), one assessed an intervention that aimed to improve the recruitment of GP practices to trials (54 practices), and one assessed an intervention that aimed to improve recruitment of GPs (150 GPs). 23 studies reported data on cost per recruit. Study size ranged between 14 and 5887 participants.

The design of included studies varied substantially and did not always fit into conventional design categories. Most studies (82/92) were what we describe as ‘yield’ studies. These types of studies appear not to have been planned as a method aiming to rigorously evaluate a recruitment intervention or interventions; rather, authors retrospectively report what methods have been used to recruit participants into the trial. Reporting of yield studies tends to rely on self-report by participants, although where online methods were used, the calculation of participant yield was recorded by the software or website; e.g. via number of clicks recorded by Facebook.

The remaining study types included in this review included cohort (7/92) and before and after designs (3/92).

### Using risk of bias to select studies

Of the 92 included studies, 72 were classified as at a ‘moderate’ or ‘serious’ risk of bias in one of more domains as well as the bias due to confounding domain. The remaining 20 studies were at ‘serious’ risk of bias in the confounding domain but were deemed to be at ‘low’ risk of bias across all other domains (participant selection, intervention classification, deviations from intervention, missing data, outcome measurement, selection of reported result).

We made the decision to focus on the 20 studies at least risk of bias in the Results and Discussion sections of this review. Primarily, this reflects our confidence in the evidence presented and follows a similar approach taken in the 2018 Cochrane recruitment review ^3^. Data from the 72 studies that we have chosen not to focus on are presented in Extended Data File 5 Characteristics of included studies ^6^. The 20 studies that we focus on here are organised into seven broad intervention categories (full references to these studies are presented in Extended Data File 6 References for the 20 studies included in the results section of this review ^6^).

The seven intervention categories are:

- Face to face recruitment initiatives
- Postal invitations and responses
- Language adaptations
- Randomisation methods
- Trial awareness strategies aimed at the recruitee
- Trial awareness strategies aimed at the recruiter
- Use of networks and databases

Due to the nature of the studies included in this synthesis, the above intervention categories frequently overlap to some extent within one study. Where lines between categories were not clear or distinct, we placed studies according to the emphasis given by the original study authors.

Table 1 shows the number of participants, GPs, and practices recruited as a result of various interventions across the seven categories for the 20 studies. This table should be viewed with caution because the general lack of denominators means that direct and meaningful comparison within and across categories is not possible, as described below.

**Table 1.** Number of participants, GPs, and practices recruited as a result of various interventions across seven categories, and spanning 20 studies.

Study	Face to face recruitment initiatives	Language adaptations	Postal invitations and responses	Randomisation methods	Trial awareness strategies aimed at the recruitee	Trial awareness strategies aimed at the recruiter	Use of networks and databased	Total number of participants (pts) /GPs/practices recruited
Andersen 2010	87		100					187 pts
Barrera 2014		A: 563 B: 2012						2575 pts
Beauharnais 2012						A: 20		31 pts
						B: 11		
Bell-Syer 2000	104		83					187 pts
Bhar 2013	27		6					33 pts
Brealey 2007				A: 322				553 pts
				B: 231				
Carr 2010					69	0		69 pts
Carter 2015	72		35		13			120 pts
Colwell 2012						A: 36		54 GP practices
						B1: 11		
						B2: 7		
Elley 2007	90		222					312 pts
Embi 2005						A: 35		59 pts
						B: 24		
Funk 2012			A: 161 B: 308					469 pts
Gill 2001	101		87					188 pts
Johnson 2015	4		A: 28		5			89 pts
			B: 27					
			C: 14					
	11 from ‘unknown non-targeted’ methods							
Lamont 2010	A: 1188 B: 1181 C: 339							2708 pts
Sawhney 2014					A: 77.7%			212 pts
					B: 45.0%			
Shah 2014	A: 32 B: 84 C: 34							150 GPs
Treweek 2010	9		9			11		29 pts
Park 2007							A: 254	442 pts
							B: 188	
Weng 2010							A: 30	44 pts
							B: 14	

Letters, e.g. ‘A’, refer to more than one intervention tested within the same intervention category. Numbers, e.g. ‘B1’, refer to instances where the same intervention was tested more than once within a study.

***Comparing data within categories***. The details of the interventions evaluated in these studies are limited, so to bring order to the variety of interventions, we have assigned them to broad categories. Each of these categories includes a range of interventions, the majority of which we are unable to thoroughly describe. For this reason, we urge you not to compare data within categories. By this we mean looking at two studies, e.g. Andersen 2010 and Bell-Syer 2000, seeing that 87 out of 187 participants and 104 out of 187 participants were recruited using face to face recruitment initiatives respectively, and assuming that these values demonstrate the success or failure of specific face to face recruitment initiatives.

We have simply used categories to bring order to the variety of interventions included in this review; each category includes a diverse range of interventions. This diversity in interventions means that none of the data presented have been pooled, and it is important that caution is exerted when interpreting data to ensure that we do not assign influence to studies where they are not deserving of it.

***Comparing data across categories***. Similarly, we urge you not to compare data across categories. By this, we mean looking at a study, e.g. Andersen 2010, seeing the 87 participants were recruited using face to face recruitment initiatives, and 100 participants were recruited using postal invitations and responses, and making a judgement about the success (or failure) of either of the interventions used. It’s important to bear in mind that these data do not provide denominators; there is no way for us to know how many people were exposed to either of these interventions, or over what time period, in order to recruit 187 participants.

### Face to face recruitment initiatives

Ten studies (totalling 3853 participants and 150 GPs) evaluated face to face recruitment initiatives, two of which used cohort studies, and eight used yield studies (see Table 1 and Table 2).

**Table 2.** Details of studies including: methods, total number of participants, GPs, and practices recruited, setting and participant population, intervention categories and comparisons.

Study	Methods	Total number of participants (pts) /GPs/practices recruited	Cost per recruit	Setting and participant population	Intervention categories	Comparisons
Andersen 2010	Yield study	187 pts	Not reported	Primary care, Norway. Adults aged 65 years or older, with a Mini-Mental State Examination (MMSE) sum score of ≥10 and ≤30 points	Face to face recruitment initiatives Postal invitations and responses	Compared the use of mailed invitation letters with face to face recruitment through routine GP practice appointments
Barrera 2014	Yield study	2575 pts	Not reported	Online, USA. Pregnant adult women aged 18 years or older	Language adaptations	Compared the use of Google AdWords in Spanish and English
Beauharnais 2012	Before and after study	31 pts	Not reported	Secondary care, USA. Patients with an HbA1C value of >7.5% who had been admitted to acute general medical or surgical units for reasons other than hyperglycaemia	Trial awareness strategies aimed at the recruiter	Compared use of an automated pre-screening algorithm with manual chart review
Bell-Syer 2000	Cohort study	187 pts	Not reported	Primary care, UK. Adults aged 18 to 60 who had experienced low back pain for no more than six months	Face to face recruitment initiatives Postal invitations and responses	Compared the use of computerised, postal referral, with manual, personal participant referral methods
Bhar 2013	Yield study	33 pts	Not reported for referral from primary care physicians or referral of inpatients. Referral of outpatients and patients at a veterans’ affairs medical centre: USD$166 and USD$44, respectively Mailing from primary care patient lists: USD$636	Primary and secondary care, USA. Adult men aged 60 or older, with a scored greater than 0 on the fourth item on the Scale for Suicide Ideation	Face to face recruitment initiatives Postal invitations and responses	Compared recruitment via referrals from primary care physicians, psychiatry residents (outpatients), an inpatient psychiatric unit, and a Veterans Affairs medical centre, and mailing from primary care patient lists
Brealey 2007	Yield study	553 pts	Not reported	Primary care, UK. Aged between 18 and 55 years inclusive, and their GP was considering referral to an orthopaedic specialist for suspected internal derangement of the knee	Randomisation methods	Compared the use of postal and telephone randomisation methods (this was deemed to be a recruitment method as delays to the start of the recruitment period were the driving force behind this change to randomisation methods).
Carr 2010	Yield study	69 pts	Community outreach event: USD$55.18 The total cost of the education event was USD$3,372.80; no participants were recruited over the subsequent four-month period	Primary care and within the community, USA. Cognitively normal and cognitively-impaired elderly people	Trial awareness strategies aimed at the recruitee Trial awareness strategies aimed at the recruiter	Compared a community outreach event aiming to improve public awareness of the trial, with a continuing medical education event aiming to improve trial knowledge among community physicians
Carter 2015	Yield study	120 pts	Face to face recruitment initiatives: GBP£105 Postal methods: GBP£15 Traditional awareness strategies: GBP£55	Primary and secondary care, and within the community, UK. Multiple sclerosis patients between 18 and 65 years of age	Face to face recruitment initiatives Postal invitations and responses Trial awareness strategies aimed at the recruitee	Compared consultant referral at MS outpatient clinics, with mail outs, and traditional trial awareness strategies
Colwell 2012	Cohort study	54 practices	Not reported	Primary care, UK. Paper reports recruitment of GP practices who were then charged with recruiting participants with type II diabetes mellitus	Trial awareness strategies aimed at the recruiter	Investigated the effect of viral marketing techniques (primer postcard and flyer) sent before invitation packs
Elley 2007	Cohort study	312 pts	Not reported	Primary care, New Zealand. Adults aged 75 of over (over 55 years for Maori and Pacific people) that had fallen in the last 12 months	Face to face recruitment initiatives Postal invitations and responses	Compared recruitment conducted in practice waiting rooms with mailed invitation letters
Embi 2005	Before and after study	59 pts	Not reported	Secondary care, USA. The paper reports on endocrinologists and general internists (main campus and community health centres) who were recruiting patients with type 2 diabetes	Trial awareness strategies aimed at the recruiter	Compared a clinical trial alert system on the electronic health record of eligible patients with traditional recruitment methods only
Funk 2012	Yield study	469 pts	Not reported	Primary and secondary care, USA. Adults over the age of 30 with a body mass index between 30 and 50 kg/m ^2^	Postal invitations and responses	Compared the method of response (telephone versus website) after potential participants were mailed a brochure about the trial
Gill 2001	Yield study	188 pts	Face to face initiatives: USD$868 Postal methods: USD$764	Primary care, USA. Adults aged 75 or older, who were physically frail	Face to face recruitment initiatives Postal invitations and responses	Compared face to face screening in primary care practices with face to face screening in the potential participant’s home
Johnson 2015	Yield study	89 pts	Not reported	Primary and secondary care, and within the community, USA. Adults with veteran status, a BMI ≥25kg/m ^2^, and a current prescription for insulin or oral medications to control blood glucose	Face to face recruitment initiatives Postal invitations and responses Trial awareness strategies aimed at the recruitee	Compared the method of response (telephone versus postcard) by potential participants after they were mailed a brochure about the trial, with staff contacting potential participants by telephone after they were mailed a brochure about the trial, as well as non-targeted flyers and clinician referrals
Lamont 2010	Yield study	2708 pts	Not reported	Primary and secondary care, and within academic centres and community affiliates, USA. Lung cancer patients	Face to face recruitment initiatives	Compared recruitment by setting; academic, community and Veterans Health Administration sites.
Sawhney 2014	Before and after study	212 pts	Not reported	Secondary care, UK. Defibrillator patients attending the cardiac arrhythmia clinic at St Bartholomew’s Hospital	Trial awareness strategies aimed at the recruitee	Compared telephone contact with participants that had been mailed information about the trial prior to their clinic appointment
Shah 2014	Cohort study	150 GPs	Not reported	Primary care, Australia. Paper reports recruitment of general practitioners by the research team	Face to face recruitment initiatives	Compared the effect of different individuals approaching general practitioners (staff from independent GP research organisation, Chief Investigator, project staff from the University)
Treweek 2010	Yield study	29 pts	Not reported	Primary Care, UK. Cohort 1: aged between 35 and 80 years, with an HbA1C value of >7% and <10%, with no prescription for any diabetes therapy in previous six months. Cohort 2: aged ≥35 and <80, with an HbA1C value of >7% and ≤9%, with a prescription for metformin within the last six months	Face to face recruitment initiatives Postal invitations and responses Trial awareness strategies aimed at the recruiter	Compared SARMA software (screening software that flags eligible patients to staff) with referral during clinic appointments, and a research nurse approaching patients at routine clinic appointments
Park 2007	Yield study	442 pts	Not reported	Secondary care and within the community, USA. Pregnant women aged 18 or older, that had smoked ≥1 cigarette in the past seven days and were up to 26 weeks of gestation	Use of networks and databases	Compared centralised recruitment efforts (i.e. existing identification and referral systems) with a de-centralised practice-based recruitment strategy (i.e. referral systems built specifically for the study)
Weng 2010	Yield study	44 pts	Not reported	Primary and secondary care, USA. Adults aged 50 or older with type 2 diabetes mellitus, an A1C measurement of between 6.5 and 8%, and pre- existing ischaemic vascular disease	Use of networks and databases	Compared a clinical data warehouse from a large medical centre, with use of a registry to identify potential participants

Face to face recruitment initiatives varied across the ten studies in this category; largely they focussed on recruitment of participants who were attending appointments with their primary care physician or GP, other studies looked at recruiting participants who were in the waiting room of their care-provider before an appointment took place. In most cases, waiting room recruitment was facilitated by a research nurse. Other methods used include referral of participants from different parts of their own clinical care pathway, though most were targeted around an existing appointment made by the potential participant. These care pathways included outpatient appointments, appointments at community institutions, academic institutions, and at veterans’ health administration centres.

Despite the superficial similarity of the interventions used within this category, both the diversity of comparators, settings and populations, and the poor reporting of the specifics of the interventions, made pooling data unfeasible.

### Language adaptations

One study (2575 participants) evaluated language adaptations; Barrera 2014 compared translations of Google AdWords in Spanish or English language using a yield study (see Table 1 and Table 2). The trial was based online within the USA and aimed to recruit pregnant women to a trial of an internet intervention for postpartum depression, the embedded recruitment study did not account for variations in how common postpartum depression is in Spanish-speaking populations in comparison to English-speaking populations.

### Postal invitations and responses

Nine studies (totalling 1614 participants) evaluated postal invitations and responses, two of which used cohort studies, and the other seven used yield studies (see Table 1 and Table 2).

Postal invitations and responses were used widely within the studies included in this review. Largely interventions within this category were based on patient lists held by caregivers; letters were sent out and then the number of responses from potential participants monitored, in most cases these studies reported the number of responses from people that ultimately went on to be recruited into the study. As mentioned in the ‘face to face recruitment initiatives’ section, many of the postal interventions used a face to face method as their comparator. Despite the superficial similarity of the interventions used within this category, both the diversity of their comparators, settings and populations, and the poor reporting of the specifics of the interventions, made pooling data unfeasible. In only one case (Funk 2010), did comparators vary from this trend. In this study, the method of response to a mailed brochure was monitored; potential participants were given the option of responding to the mailing by telephone or a website. These comparators were unusual within the literature, and draw attention to the two-dimensional nature of many of the other studies within this category; largely researchers looking at postal methods are focussing on the method used to contact potential participants, rather than the ways that these individuals may respond.

### Randomisation methods

One study (553 participants) evaluated randomisation methods; Brealey 2007 compared use of telephone and postal randomisation methods using a yield study (see Table 1 and Table 2). Initially, general practices involved used a telephone service to randomise patients to the host trial. Delays in the start of recruitment at some sites led the team to modify the randomisation procedure to include postal randomisation. Following this, new sites were given the option to use either postal or telephone randomisation methods.

### Trial awareness strategies aimed at the recruitee

Four studies (totalling 407 participants) evaluated trial awareness strategies aimed at the recruitee, one of which used a before and after study, and the remaining three used yield studies (see Table 1 and Table 2).

This category is diverse; the four studies include four distinct interventions. The reporting of these interventions is ambiguous; for example, Carr 2010 describes a community outreach event, Johnson 2015 describes a non-targeted flyer, and Sawhney 2014 describes increased awareness of the trial via use of a telephone reminder prior to their clinic appointment. It is feasible that all of these interventions could come under the umbrella of ‘trial awareness strategies aimed at the recruitee’ which is what is described by Carter 2015. The text states that Carter 2015’s interventions included distribution of leaflets and posters at clinics, therapy centres and regional multiple sclerosis societies, presentations and attendance at regional multiple sclerosis events and to local physiotherapy teams, and referral from other professional such as multiple sclerosis nurses and word of mouth.

### Trial awareness strategies aimed at the recruiter

Fives studies (totalling 188 participants and 54 practices) evaluated trial awareness strategies aimed at the recruiter, one of which used a cohort study, two used before and after studies, and two used yield studies (see Table 1 and Table 2).

Again, the interventions evaluated within this category are diverse: Carr 2010 looked at a medical education event; Embi 2005 and Treweek 2010 looked at methods of clinical trial alert software set to trigger during clinic appointments; Beauharnais 2012 assessed effectiveness of an automated pre-screening algorithm to identify potential participants; and Colwell 2012 evaluated the use of viral marketing techniques in the form of postcards, invitation letters and flyers. The diversity of these interventions means that data could not be pooled.

### Use of networks and databases

Two studies (totalling 486 participants) evaluated the use of networks and databases, both of which used yield studies (see Table 1 and Table 2).

Park 2007 compared centralised recruitment efforts with de-centralised approaches that were tailored to the study and sites specifically. Weng 2010 evaluated effectiveness of existing lists of potentially eligible participants; comparing a clinical patient registry with a clinical data warehouse. The interventions are sufficiently different that data could not be pooled.

## Discussion

This review identified 92 studies, 20 of which were included in a narrative synthesis; those 20 studies evaluated the effect of seven categories of interventions to improve recruitment to randomised trials.

The interventions evaluated in these studies varied significantly; even those that had an intervention category in common were sufficiently dissimilar to prevent pooling of data, rendering sub-group analyses unfeasible. That said, what limits the utility of these studies is not necessarily the interventions evaluated; it is the abundance of small study samples sizes, inadequate reporting, and a lack of coordination when it comes to deciding what to evaluate and how.

### Does this mean that non-randomised evaluations of recruitment should be stopped in favour of implementing randomised approaches?

This review does not show ground-breaking evidence that will change the global landscape of how trialists recruit participants into trials. However, the 2018 Cochrane recruitment review ^3^ of randomised evaluations of recruitment interventions was not able to provide clear evidence of benefit for the majority of interventions either. Like this review, the randomised review also experienced challenges with small, methodologically flawed studies, a diverse range of interventions, and a lack of detailed reporting. This fact may not be comforting for trialists, but it demonstrates that the utility of non-randomised studies is not always vastly different from their randomised counterparts.

Non-randomised evaluations have acquired a bad reputation, but they do have their merits. Randomised evaluations are not always possible because of logistics, financial resources, or ethical reasons ^10^, and non-randomised studies could allow researchers to gather useful data to complement or replace data generated by randomised trials ^11^.

It is clear that non-randomised evaluations of recruitment interventions will continue. In their current form, however, we found their usefulness to others to be extremely limited. What we need to focus on now is improving the way that these non-randomised evaluations are planned, conducted and reported.

### Planning non-randomised evaluations of recruitment

The non-randomised studies that are included in this review largely take the form of what we refer to as ‘yield’ studies. As described earlier, these types of studies appear not to have been planned as a means to rigorously evaluate a recruitment method; instead, they represent the work of authors retrospectively reporting what they have done, and subsequently what they have seen.

This practice limits utility of these studies in two ways:

1. The studies are not designed in such a way as to lend themselves to straightforward analysis, which means that interventions and their comparator are not always introduced at the same time or used for the same length of time. A lack of planning also results in the collection of data that are incomplete and lack context; this is a problem that features in most studies included in this review. Data are presented in terms of numerators; they provide numbers of participants/GPs/practices recruited into a trial, but do not provide a denominator, meaning that comparing interventions to assess effectiveness is impossible.
2. As is clear from the larger intervention categories such as face to face recruitment initiatives, and postal invitations and responses, the trials community is currently lacking a consistent approach to the non-randomised evaluations that they are publishing.

Rather than reporting what has been done retrospectively, we would encourage trialists to prospectively plan to embed recruitment evaluations, specifically using a study within a trial (SWAT) protocol ^12^ that already exists on the SWAT repository ^13^, into their trials from the very beginning of the process of planning the host trial. The Medical Research Council Systematic Techniques for Assisting Recruitment to Trials (START) project is a remarkable example of the effectiveness of a well-planned, organised and cohesive approach to SWATs ^14^; the project ran between 2009 and 2015 and answered its research question regarding optimised participant information sheets within the space of six years. The follow-on PROMETHEUS project is co-ordinating over 30 recruitment and retention SWATs and will substantially increase global evidence for trial recruitment and retention in the space of around four years. Without coordination of high-quality evaluations, it is entirely possible for a decade to pass without materially increasing the evidence base available to trialists, as a comparison of the 2007 ^15^ and 2018 ^3^ Cochrane recruitment reviews demonstrates.

### Conducting non-randomised evaluations of recruitment

The process of conducting non-randomised evaluations of recruitment lacks structure; limited planning means that many of the studies included in this review were penalised as a result of poor conduct.

74% of included studies were judged to be at moderate risk of bias in the ‘bias in classification of interventions’ domain of the ROBINS-I tool. These studies were most often penalised as a result of blurred lines between interventions and their comparators. For example, Adams 1997 (Extended Data File 5 Characteristics of included studies ^6^) compares the effectiveness of professional referrals, cold calling by the research team, presentations at senior centres, media outreach, mailings sent to personal care home managers, and flyers; a total of six interventions. Participants could conceivably have been drawn to take part in the trial as a result of more than one of these six interventions; someone could have seen the media outreach campaign, received a flyer, *and* attended a presentation at a senior centre. This, combined with self-report of one method by participants, makes meaningful interpretation of the results extremely difficult.

### Reporting non-randomised evaluations of recruitment

Currently, trialists are focussing on the mode of delivery of the interventions that they are working to evaluate; they omit key details regarding the content of the intervention, as well as the specific timescales that interventions were in place for. We highly encourage the use of the Guidelines for Reporting Non-Randomised Studies ^16^, and the Template for Intervention Description and Replication (TIDieR) checklist and guide ^17^ when reporting these types of studies.

Missing data was another aspect of reporting where detail was lacking. Of the 92 included studies, 13% were deemed to be at serious risk of bias due to missing data; a glaring example of research waste. Pieces of data that were missing were not entire data categories or a reflection of participants being lost to follow-up; in some cases, the data simply did not add up. One example is Blackwell 2011; this paper reported recruitment of 301 participants, but when we manually calculated how many participants had been recruited across each of the seven methods used in the study, and also included the participants that were reported as ‘don’t know/refused/other’, the total was 303 participants. The size of the discrepancy may appear trivial, but it undermines confidence in the data presented and the study generally. This was not a unique occurrence; missing data were also found in Brownstone 2012 ^18^, Freret 2003 ^19^, Kernan 2009 ^20^, Lewis 1998 ^21^, Martin 2011 ^22^, McDermott 2009 ^23^, Piantadosi 2015 ^24^, Silagy 1991 ^25^, Tenorio 2011 ^26^, Unlü Ince 2014 ^27^, and Zhou 2013 ^28^. If non-randomised evaluations of recruitment interventions are to have any value, how they are reported needs to improve.

## Conclusions

### Implications for systematic reviews and evaluations of healthcare

Some interventions to increase recruitment described in this review do show promise but methodological and reporting problems mean that our confidence in these results is not substantial enough to recommend changes to current recruitment practice. Currently the literature is oversaturated with a diversity of interventions tested in non-randomised evaluations that fail to drill down deep into the effects of each specific recruitment strategy. Their usefulness to other trialists is therefore extremely limited.

What is needed now is a move away from retrospective descriptions of what happened, to carefully planned prospective evaluations of well-described recruitment interventions and their comparators. Without this change, authors of non-randomised evaluations of recruitment interventions are simply contributing to research waste.

## Data availability

### Underlying data

All data underlying the results are available as part of the article and no additional source data are required.

### Extended data

Open Science Framework: A systematic review of non-randomised evaluations of strategies to improve recruitment to randomised controlled trials. https://doi.org/10.17605/OSF.IO/98BQ4 ^6^

This project contains the following extended data:

- Extended Data File 1 Blank data extraction form.docx
- Extended Data File 2 References to studies awaiting assessment.docx
- Extended Data File 3 Characteristics of excluded studies.docx
- Extended Data File 4 Studies that were at a critical risk of bias and therefore excluded from this review.docx
- Extended Data File 5 Characteristics of included studies.docx
- Extended Data File 6 References for the 20 studies included in the results section of this review.docx

### Reporting guidelines

Open Science Framework: A systematic review of non-randomised evaluations of strategies to improve recruitment to randomised controlled trials. https://doi.org/10.17605/OSF.IO/98BQ4 ^6^

Data are available under the terms of the Creative Commons Attribution 4.0 International license (CC-BY 4.0).
