# Supplementary material for: A systematic review of non-randomised evaluations of strategies to improve participant recruitment to randomised controlled trials
Source: F1000Res. 2020 Feb 5;9:86. [Version 1] doi: 10.12688/f1000research.22182.1 (PMC7336048; doi:10.12688/f1000research.22182.1)
Supplement: Peer review history

# Peer review: Reviewer response for version 1

**DOI:** 10.5256/f1000research.24467.r65425

**Published:** 2020-07-10

**Authors:** Lucy Beasant

**Affiliations:** 1 Centre for Academic Child Health, Bristol Medical School, University of Bristol, Bristol, UK

Thank you for requesting that I review this interesting and timely article, I found it very informative. Interventions that enhance recruitment to non-randomised research studies are an important and under reported area, this systematic review highlights the lack of clear, reliable evidence presented to date. This is an important article because it highlights methodological and reporting problems, whilst at the same time bringing together evidence from interventions used in non-randomised studies that do show ‘promise’ in terms of improving recruitment. In addition the article discusses and highlights the way in which future non-randomised studies might improve reporting (and conduct) of similar recruitment interventions, enabling researchers to investigate and utilise effective recruitment strategies during the planning and implementation stages of research studies. In my view this systematic review has been conducted with methodological rigour, however, I am a qualitative researcher/trialist and trust that this article will also be reviewed by a statistician/ systematic reviewer who routinely uses the ROBINS-I tool to assess for risk of bias. The authors have provided clear and explicit detail in relation to the studies and intervention categorisation used for the narrative synthesis of data. I believe this is a very strong piece of work in its current format and would recommend indexing.

Are the rationale for, and objectives of, the Systematic Review clearly stated?

Yes

Is the statistical analysis and its interpretation appropriate?

I cannot comment. A qualified statistician is required.

Are sufficient details of the methods and analysis provided to allow replication by others?

Yes

Are the conclusions drawn adequately supported by the results presented in the review?

Yes

Reviewer Expertise:

Qualitative Researcher: Randomised Controlled Trials, Equipoise, Recruitment and Retention.

I confirm that I have read this submission and believe that I have an appropriate level of expertise to confirm that it is of an acceptable scientific standard.

# Peer review: Reviewer response for version 1

**DOI:** 10.5256/f1000research.24467.r65424

**Published:** 2020-07-08

**Authors:** Geoff K Frampton

**Affiliations:** 1 Southampton Health Technology Assessments Centre, Wessex Institute, University of Southampton, Southampton, UK

This article reports on a systematic review of non-randomised studies that have evaluated approaches for improving the recruitment of people into randomised controlled trials (RCTs). The objective was “to quantify the effects of strategies to improve participant recruitment to randomised trials evaluated using non-randomised study designs”. The authors acknowledge that “currently, non-randomised evaluations of trial recruitment interventions are rejected due to poor methodological quality”, but their rationale for conducting this review is that “without collation, this body of evidence is currently being ignored, and may hold substantial/promising undiscovered effects”.

 This is an important area of research since recruitment into RCTs is challenging; and failed or suboptimal trial recruitment often leads to trials being abandoned, creating a substantial amount of research waste, as well as having negative effects on trial participants and delaying the uptake of clinical trial findings into policy and practice.

**General comments:**

    I have read this paper with interest because most studies on trial recruitment are non-randomised and it would, therefore, be helpful to obtain as much useful information as possible from these studies to inform research practice and policy. However, a major obstacle, as the authors acknowledge, is the poor methodological quality of non-randomised recruitment studies. According to the stated rationale for doing this work I was expecting this systematic review to make the best use of the available data that could be extracted from these methodologically poor studies, thereby providing an advance on previous research. For instance, the methods section suggests that GRADE methods might enable methodologically weak studies to contribute informatively to data synthesis. But this review did not explore any methods such as GRADE that might have enabled any of the data from these studies to contribute to a comparative analysis. And the review stops short of providing any comparative analysis, thereby failing to address its stated objective. I assume that this is because the included studies were deemed too weak to be useful, although this is not explicitly stated or discussed. I agree with the authors that the statistical pooling of studies' results would not have been appropriate, due to the heterogeneity of study designs. A narrative synthesis would, therefore, be reasonable, as the authors acknowledge. However, no integration of the comparative results, strengths, and limitations of any of the individual studies is presented, meaning that effectively there is no narrative synthesis (though a summary of some study characteristics is given).

    I appreciate that it is not easy to do a systematic review on this topic area due to the considerable heterogeneity and weaknesses of the studies, so I sympathise with the authors. But I am surprised that some of the problems with this evidence base were not identified during the protocol development, scoping, and pilot testing of the methods. The upshot is that very weak (and seemingly useless) studies appear to have made it all the way through to the data synthesis step but then all of them were rejected from narrative synthesis. Could the evidence synthesis planning process have been done differently perhaps, to identify some of the methodological problems (e.g. limited sample sizes and ambiguous descriptions) earlier, or would this have just resulted in all studies being excluded? It feels to me that all studies have, effectively, been excluded anyway since none of them provide any comparative results to support the review objective. Are there any lessons that review teams can learn here, to improve future evidence syntheses in this difficult area?

     A comprehensive risk of bias assessment was conducted, but this is not reported consistently. For example, the 7 individual domains of bias are reported for the 10 studies that were excluded because they were at critical risk of bias (Extended data file 4) but only an overall risk of bias judgement is reported for the 72 studies that were included in the review (Extended data file 5). Given that a total of 92 studies were eligible for inclusion in the review, the risk of bias assessments for 10 studies appear to be missing (Extended data files 4 and 5 report 82 studies in total).

    The review report is not fully clear about which of the methodological issues discussed were assessed as risks of bias, and which were identified additionally. Missing data are described separately in the Discussion (e.g. for the Blackwell 2011 study) but I presume that these missing data had already been captured in the “Missing data” domain of the risk of bias assessment? It would be helpful if each of the risk of bias domains could be explained so that their interpretation in relation to non-randomised recruitment studies is clear.  

    In summary, this review has failed to meet its stated objective and it is difficult to see how it advances knowledge in this scientific area (we already knew that non-randomised studies are methodologically weak, and the rationale behind this review was to make best use of these studies despite their limitations... which hasn't been achieved). 

    But I believe the authors could update this manuscript to make it into a useful scientific contribution. I have suggested two possibilities that the authors could consider. I am not sure how much effort these would take but they would utilise the studies already included in the review and may require little if any amendment to the current protocol:

1. Check the included studies and evaluate systematically whether there are any data that can be obtained from these non-randomised studies that would constructively inform a data synthesis (i.e. go back and attempt to fulfill the stated objective). Currently, the review considers only limitations, whereas it would be helpful to look for the strengths (if any) that can be taken from these non-randomised studies. Would GRADE or other approaches (e.g. sensitivity analyses) enable any of the included studies to contribute to data synthesis? Could you make best- / worst-case assumptions in sensitivity analyses to address some of the uncertainties? (see specific comments below). I would be surprised if all 92 identified studies are so poor that none of them at all can be descriptively reported in the narrative synthesis, but this should be systematically checked and transparently reported so that if it is true that the entire evidence base is useless then at least the conclusion would be defensible.
2. If the evidence-based conclusion is that data synthesis is not feasible due to all studies being too weak, then pragmatically the review could direct its effort to systematically analysing the limitations of the studies as a basis to support evidence-based recommendations on how to improve non-randomised studies in future. In order to make the critique of the studies’ methods more systematic, consistent, and transparent perhaps a table could be provided in the results section for all the included studies indicating exactly which methodological limitations each of the studies was susceptible to? This could then support evidence-based specific recommendations about exactly what needs to be improved in the non-randomised studies for research practice to be improved. I think this could make the paper valuable – provided that the recommendations arising are evidence-based and are communicated and disseminated carefully to achieve impact.

 

**Specific comments:**

1. Methods: Types of intervention: I don’t understand the meaning of the following sentence (NB minor typo needs correcting): “Any intervention or approach aimed at improving or supporting recruitment of participants nested within studies performed for purposed unrelated to recruitment.”
2. Methods: Search methods for identification of studies: “Adding these studies into the review would not strengthen or disprove the conclusions we had already drawn.” This statement seems to imply that the 10 studies being referred to were somehow tested for their influence on the overall results. I suspect this is not the intended meaning. Do you mean that, by virtue of their lack of clarity, these studies could not usefully inform the review?
3. Methods: Assessment of risk of bias in individual studies: Very little description or discussion of the risk of bias assessment is provided. As ROBINS-I is a relatively new tool I wonder how many readers will be familiar with it? Could a supporting explanation be provided of how decisions were reached? If the review is re-framed to focus on the studies’ weaknesses (see my general comments above), then a more detailed discussion of risks of bias would be appropriate. How good was reviewer agreement on rating the risks of bias? Were any specific bias domains particularly difficult to interpret or agree on?
4. Figure 1: PRISMA chart: What were the reasons that so many (33) full-text articles were not available? Is there anything practical that could be recommended to reduce this problem in future research?
5. Results: Screening and identification of studies: “this includes seven articles which required additional data to allow for inclusion”. The meaning of this statement is not clear. Do you mean that insufficient information was reported in these studies to make an eligibility decision and since the authors did not provide any further information these studies had to be excluded?
6. Results: Using risk of bias to select studies: The meanings of the seven bullets listed here are not explicitly defined anywhere, although to me they seem intuitive and self-evident. An exception is “Language adaptations” which I didn’t understand the meaning of until I reached Table 2, where I noticed that it is explained (implicitly). Perhaps cross-refer the reader to Table 2 to clarify what these bullets mean?  
7. Table 8: Minor typo in the heading of Column 8.
8. Results: Postal invitations and responses: “As mentioned in the ‘face to face recruitment initiatives’ section, many of the postal interventions used a face to face method as their comparator”. This statement might be misleading as the text in the Face to face recruitment initiatives section does not say anything about postal interventions. However, the information referred to is in Table 2, so perhaps refer directly to the table? For studies that spanned these two categories, how was a decision made on which category to allocate the study to?
9. Results: Trial awareness strategies aimed at the recruitee: Minor typo: “professional”.
10. Results: Trial awareness strategies aimed at the recruiter: Minor typo: “fives”.
11. Discussion: “Non-randomised evaluations have acquired a bad reputation, but they do have their merits. Randomised evaluations are not always possible because of logistics, financial resources, or ethical reasons” This statement seems to be referring to non-randomised studies in general. Are there specific reasons why some (or indeed most) types of recruitment study can only be non-randomised?
12. Discussion: Some information about the Adams 1997 study is revealed here but it feels like this information should have been reported in the results section, along with equivalent information from the other included studies, for consistency and transparency.
13. Discussion: “Currently, trialists are focussing on the mode of delivery of the interventions that they are working to evaluate; they omit key details regarding the content of the intervention, as well as the specific timescales that interventions were in place for.” Is this statement evidence-based? It doesn’t really link to the results section.
14. Discussion: The issue of missing data has not been consistently covered for each study in the results section, so it is unclear how representative the discussion of missing data in the Blackwell 2011 study is of the studies in general. It feels harsh to say that 2 missing data (<1%) would be sufficient to cause mistrust in the entire Blackwell 2011 study. Were such strict criteria applied across all studies? Could you do a (quantitative or descriptive) sensitivity analysis – i.e. explore whether changing the sample size by n=2 would make any tangible difference to the outcomes? I think even Cochrane Review guidance is pragmatic about not being overly strict if the amount of missing data is trivial?
15. Discussion: The review included several study designs of which Yield studies were the most frequent and, apparently, least useful. Could the remaining study types that would be more reliable tell us anything? No results from any of these studies have been provided.
16. Conclusions: The statement “Some interventions to increase recruitment described in this review do show promise” is not evidence-based, since no evaluation of intervention effectiveness has been provided.
17. References: There is no information provided for reference 15.

Are the rationale for, and objectives of, the Systematic Review clearly stated?

Yes

Is the statistical analysis and its interpretation appropriate?

Not applicable

Are sufficient details of the methods and analysis provided to allow replication by others?

Partly

Are the conclusions drawn adequately supported by the results presented in the review?

No

Reviewer Expertise:

Evidence synthesis

I confirm that I have read this submission and believe that I have an appropriate level of expertise to state that I do not consider it to be of an acceptable scientific standard, for reasons outlined above.

# Peer review: Reviewer response for version 1

**DOI:** 10.5256/f1000research.24467.r65427

**Published:** 2020-07-03

**Authors:** Emily Peckham

**Affiliations:** 1 Mental Health and Addictions Research Group, Department of Health Sciences, University of York, York, UK

Thank you for inviting me to review this systematic review of non-randomised evaluations of strategies to improve participant recruitment to RCTs. I have listed some suggestions below that I believe would improve the article. 

**Search methods:**

- Were there any date limits applied to the searches or were they from the databases inception?
- The number of additional abstracts identified after the 2018 search is given in the methods but not the number identified in the original search which is confusing. I am also not convinced by this rationale for not updating the searches. I think it would be better to update the searches and include the information from the 10 studies identified. I am not clear why this wasn't done.

**Data extraction:**

- It would be helpful to give an overview of what data was extracted. 

**Figure 1:**

- It would be helpful to list the reasons for exclusion at the full-text stage.
- It would be better to combine the included studies boxes into one, stating 'x articles were identified comprising of x unique studies' or similar

**Results:**

- I am not sure what is meant by the statement that 7 articles were excluded at the full-text stage because they needed additional data to allow for inclusion. From the information in the study was it not possible to determine whether the study was eligible? Or is it that there was some of the data you wanted to extract was missing. If it is the latter I would include the study and state that there was missing data. 

**Using risk of bias to select studies:**

- Of the 72 studies excluded what was their risk of bias due to the confounding domain, it just states 'the bias due to confounding domain'. 

**Comparing data within categories:**

- I think the following statement needs to be rephrased or removed. I am not clear why you are unable to describe the intervention. 'Each of these categories includes a range of interventions, the majority of which we are unable to thoroughly describe.' 

**Planning no-randomised studies:**

- The acronym SWAT is used for the 1st time without being spelt out fully, it is only spelt out fully later on in the paragraph. 
- It would be useful in this section to explain what a SWAT is, as it stands the section focuses on what is limiting the utility of evaluations of recruitment and it would be improved and more useful if more focus is given on what could be done to increase the utility of these studies. 

**Reporting non-randomised studies:**

- It would be helpful to give an overview of the type of data that was missing. At present this section is focusing on the fact that the number of participants didn't add up in some of the studies. Was this the main example of missingness? 

Are the rationale for, and objectives of, the Systematic Review clearly stated?

Yes

Is the statistical analysis and its interpretation appropriate?

Not applicable

Are sufficient details of the methods and analysis provided to allow replication by others?

Partly

Are the conclusions drawn adequately supported by the results presented in the review?

Yes

Reviewer Expertise:

Severe mental illness, smoking cessation, RCTs

I confirm that I have read this submission and believe that I have an appropriate level of expertise to confirm that it is of an acceptable scientific standard, however I have significant reservations, as outlined above.

# Peer review: Reviewer response for version 1

**DOI:** 10.5256/f1000research.24467.r65416

**Published:** 2020-06-30

**Authors:** Barbara Clyne

**Affiliations:** 1 HRB Centre for Primary Care Research, Department of General Practice, Royal College of Surgeons in Ireland, Dublin, Ireland

This systematic review includes non-randomised studies of strategies to improve participant recruitment to randomised controlled trials. The justification for inclusion of non-randomised studies is that the previous Cochrane systematic review of RCTs found very little high-certainty evidence to guide trialists. This is a well presented, large scale, robust systematic review of a topic of great importance to trialists seeking strategies to improve their trial’s recruitment.

 The inclusion of non-randomised studies resulted in a diverse number of evaluations and the narrative synthesis is as a result quite broad and high-level. The recommendations for use of TIDIER in intervention reporting and SWATS are practical and feasible. There are a number of clarifications that I feel would improve the manuscript overall. These are as follows:

1. In the introduction could you please give some detail as to what strategies were identified as effective from the Cochrane review. In the discussion can you compare and contrast the findings of this current review to those of the Cochrane review. It would be helpful to get an understanding of what the addition of these non-randomised studies adds overall to the evidence base, given the substantial amount of work in this review. Included studies were of insufficient quality to allow for the pooling of data, however, the authors could present narratively a summary of strategies that demonstrated success, to guide trialists, and those designing SWATS as to what strategies may be worth exploring further.   
2. The literature search was carried out in 2015. The authors have justified this by including details of how a search in 2018 identified an additional 10 studies only, the addition of which would not strengthen or disprove the conclusions. However, this search is 5 years out of date and I think the authors should contextualise their conclusions in light of this limitation. The addition of more ‘yield studies’ would be unlikely to change the conclusions (as these would be likely high risk of bias and not included in the analysis), however, the addition of a few more cohorts studies could have a bigger impact.
3. Were all non-yield studies pre-planned studies?
4. The review identified 92 studies, but only 20 were considered low risk of bias and included in the narrative review. Could the authors comment on 20 included studies in more detail in the manuscript? For example, from my reading for the table, this includes 13 yield studies and 7 cohorts, etc. type studies.
5. Could the authors comment on the definition of non-randomised study – I wonder if a more detailed definition was applied would this have reduced the number of yield studies that were identified?
6. In the introduction, the authors comment that aggregating data from non-randomised studies using the Grading of Recommendations Assessment, Development and Evaluation (GRADE) approach, may raise confidence in the overall body of evidence, and supplement the evidence-base from randomised studies. The authors have not used GRADE in this manuscript or commented on why they have not. There is good recent guidance on the use of GRADE with ROBINS-I available, see Schünemann HJ, Cuello C, Akl EA, et al. GRADE guidelines: 18. How ROBINS-I and other tools to assess risk of bias in nonrandomized studies should be used to rate the certainty of a body of evidence. J Clin Epidemiol. 2019;111:105-114. ^1^

Are the rationale for, and objectives of, the Systematic Review clearly stated?

Yes

Is the statistical analysis and its interpretation appropriate?

Not applicable

Are sufficient details of the methods and analysis provided to allow replication by others?

Yes

Are the conclusions drawn adequately supported by the results presented in the review?

Partly

Reviewer Expertise:

Evidence synthesis, RCTs, process evaluation

I confirm that I have read this submission and believe that I have an appropriate level of expertise to confirm that it is of an acceptable scientific standard, however I have significant reservations, as outlined above.

## References

[1] : GRADE guidelines: 18. How ROBINS-I and other tools to assess risk of bias in nonrandomized studies should be used to rate the certainty of a body of evidence. *J Clin Epidemiol*.111: 10.1016/j.jclinepi.2018.01.012 105-114 10.1016/j.jclinepi.2018.01.012 29432858PMC6692166

# Peer review: Reviewer response for version 1

**DOI:** 10.5256/f1000research.24467.r59580

**Published:** 2020-02-13

**Authors:** Karen Bracken

**Affiliations:** 1 NHMRC Clinical Trials Centre, The University of Sydney, Sydney, NSW, Australia

This systematic review is a companion to the 2018 Cochrane review of strategies to improve recruitment to clinical trials. Unlike the 2018 review, which included only randomised evaluations of strategies, this review includes only non-randomised evaluations. Given the 2018 review found that few recruitment strategies were supported by good quality evidence, this new review had the potential to build on the limited evidence base for trialists seeking strategies to improve their trial’s recruitment.

 The authors faced the challenge of synthesizing a large number of diverse evaluations and so the resulting synthesis is wide-ranging and necessarily high-level. The authors found the included studies of insufficient quality to allow for pooling of data and, as they point out, this limits the usefulness of this review in guiding trialists to select promising recruitment strategies. They mentioned in the conclusion that some interventions showed promise but it was unclear to me which specific interventions these were. This would be useful information to guide future research.

 The large number of retrospective, comparative “yield” studies included in the review is perhaps unsurprising given that many evaluations were seeking to compare strategies to invite people to take part in trials. The authors criticize these studies for reporting the numerator but not the denominator in such comparisons, for blurring lines between interventions/comparators and for lack of forward planning when designing evaluations. However, these limitations seem to reflect the real-world challenges of implementing such strategies. Even with forward planning, there seems to be no way to calculate the denominator for strategies like media outreach and flyers, and there may be few practical ways to prevent participants from being exposed to multiple strategies without causing potential harm to the host trial recruitment. Did the authors feel there was any way that such evaluations could be of value outside the trial in which they were implemented?

 The authors did suggest that recruitment evaluations would be of greater value if they reported their interventions more clearly and completely, using the TIDieR checklist. This seems like a practical suggestion and I hope it will be widely taken up.

 I found this review a thought-provoking but sobering read. The fact that such a large-scale, rigorously-conducted review resulted in not a single recommendation of a strategy to improve recruitment should be a wake-up call to all of us researching in this area.

Are the rationale for, and objectives of, the Systematic Review clearly stated?

Yes

Is the statistical analysis and its interpretation appropriate?

Yes

Are sufficient details of the methods and analysis provided to allow replication by others?

Yes

Are the conclusions drawn adequately supported by the results presented in the review?

Partly

Reviewer Expertise:

Trial operations methodology research

I confirm that I have read this submission and believe that I have an appropriate level of expertise to confirm that it is of an acceptable scientific standard.
